# Entropy Generation Analysis of the Flow Boiling in Microgravity Field

**DOI:** 10.3390/e24040569

**Published:** 2022-04-18

**Authors:** Zijian Sun, Haochun Zhang, Qi Wang, Wenbo Sun

**Affiliations:** School of Energy Science and Engineering, Harbin Institute of Technology, Harbin 150001, China; 21b902052@stu.hit.edu.cn (Z.S.); 21s002039@stu.hit.edu.cn (Q.W.); 21s102109@stu.hit.edu.cn (W.S.)

**Keywords:** numerical simulation, microgravity field, flow boiling, entropy generation, performance evaluation

## Abstract

Entropy generation analysis of the flow boiling in microgravity field is conducted in this paper. A new entropy generation model based on the flow pattern and the phase change process is developed in this study. The velocity ranges from 1 m/s to 4 m/s, and the heat flux ranges from 10,000 W/m^2^ to 50,000 W/m^2^, so as to investigate their influence on irreversibility during flow boiling in the tunnel. A phase–change model verified by the Stefan problem is employed in this paper to simulate the phase–change process in boiling. The numerical simulations are carried out on ANSYS-FLUENT. The entropy generation produced by the heat transfer, viscous dissipation, turbulent dissipation, and phase change are observed at different working conditions. Moreover, the *Be* number and a new evaluation number, *E*_P_, are introduced in this paper to investigate the performance of the boiling phenomenon. The following conclusions are obtained: (1) a high local entropy generation will be obtained when only heat conduction in vapor occurs near the hot wall, whereas a low local entropy generation will be obtained when heat conduction in water or evaporation occurs near the hot wall; (2) the entropy generation and the *Be* number are positively correlated with the heat flux, which indicates that the heat transfer entropy generation becomes the major contributor of the total entropy generation with the increase of the heat flux; (3) the transition of the boiling status shows different trends at different velocities, which affects the irreversibility in the tunnel; (4) the critical heat flux (CHF) is the optimal choice under the comprehensive consideration of the first law and the second law of the thermodynamics.

## 1. Introduction

The studies on lighter, smaller spacecraft with more functions have been promoted by deeper space exploration missions [1]. The limited volume of a highly integrated spacecraft with many heat sources brings new challenges to the thermal-control system. The challenges include a strict temperature control (±1 K), a high local heat flux (>100 W/cm^2^), extreme high temperature environments, and so on [2]. An outstanding heat transfer capacity can be obtained through the huge latent heat during the boiling phase change process at a constant temperature. The unique thermodynamic characteristics of the boiling process make it a good choice for the designs of the thermal control system. The thermal control systems based on boiling phenomenon have successfully been utilized [3,4,5] in industrial manufacturing with strict requirements for temperature. It provides a promising prospect in the design of spacecraft cooling systems, compared with the thermal control systems based on the single phase [6]. The cooling system in a spacecraft operates under a microgravity field, which is totally different from the Earth. The absence of gravity contributes to a different boiling phenomenon in space compared with that on earth. Bubbles generated when boiling tend to adhere on the hot wall under the microgravity field, distinguishing the heat and mass transfer characteristics from that under the gravity field [7].

Plenty of the studies focusing on the boiling characteristics in the microgravity field have been conducted by space agencies in different countries to obtain a reliable and efficient thermal control system. A set of experiments during two parabolic flights was carried out by Brutin [8], in which the HFE-7100 was employed as the working medium. The research was designed to explore the factors that enhance the heat transfer capacity of flow boiling in rectangular microchannels. The flow patterns were visualized by image processing in this paper to study the pressure drop and the void fraction of the flow in the rectangular microchannels. It is observed that the acceleration has great influence on the flow pattern of two-phase flow, which determines the heat transfer capacity of flow boiling in different acceleration fields. The pressure loss inside the rectangular microchannels in microgravity is half that of normal gravity in the rectangular mini-channels, and it can be observed in big slugs. A lower film thickness tends to be observed in microgravity, enhancing the boiling heat transfer capacity of the liquid. The improvement of the boiling heat transfer capacity of the liquid and the slugs in the two-phase flow may decrease the critical heat flux (CHF), putting the thermal control system at a disadvantage. Steven [9] acquired a CHF model that was modified based on the results of the International Space Station (ISS), which adopted the C6F14 as the working medium. The model acquired by Steven [9] made a set of relatively correct predictions with an uncertainty of 19.04%. Other phenomena such as the bubble dynamics and the vaporization feature are also investigated [10,11,12,13,14]; however, the aspects of the studies were mainly focused on the first law of thermodynamics, lacking the analysis that is based on the second law of thermodynamics. 

The entropy generation analysis, as a powerful tool, has been adopted to investigate the feature of a thermal system for decades [15,16,17]. The contributors to an inefficient thermal system can be found out using entropy generation analysis based on the second law of thermodynamics. The optimal design of the thermal system can be realized through the correction of the improper designs, guaranteeing the proper functions of a spacecraft. The principles of entropy generation minimization were developed by Bejan [18]. The investigation based on the principles proposed by Bejan [18] can bridge the gap between heat transfer, flow pattern, and thermal dynamics, providing a unified analysis of the multi-dimension issue [19,20]. The entropy generation analysis method has been employed in studies on the heat transfer characteristics of nanofluids, obtaining exciting achievements [21,22,23,24]. An experimental study was carried out by Abous [25,26] to investigate the entropy generation characteristics during the flow boiling in helically coiled tubes. It can be concluded from this research that the total entropy generation has a positive correlation with vapor quality, as well as the mass of the vapor. The entropy generation during flow boiling in a in a micro-fin tube was investigated by Revellin and Bonjour [27] to explore the optimal structure of the tunnel with a certain heat flux. Experimental research was conducted by Shahriya [28] to characterize the entropy generation during the flow boiling in a twisted-tape tunnel. The operating conditions were taken into consideration as well. It can be found from experimental studies that [25,26,27,28] the results obtained in the experiments are precise, but the laws acquired in this research are not universal at different operating conditions. In addition, the influence of the flow patterns, such as vapor–liquid distribution, on the local entropy generation, cannot be directly acquired due to the limitations of experimental techniques. In order to achieve a universal entropy generation law inside a semicircular channel, Y.S. [29] analyzed the entropy generation characteristics of flow boiling using an empirical formula fitted by previous studies [30], and obtained the entropy generation characteristics at different operating conditions. Hojati [31] discussed the effect of geometrical parameters and flow conditions on the entropy generation during the flow boiling of R134a in horizontal internally grooved tubes. Although the empirical formulas predict the phenomena of flow boiling to a certain degree of accuracy in terms of the first law of thermodynamics, the uncertainty inevitably introduces errors into the flow boiling entropy generation analysis. Numerical simulation is a powerful research technique to obtain more detailed parameters of flow field characteristics [19,20]. By establishing the numerical models, the simulation of the boiling behavior in the microgravity field can be obtained as well as more details of the flow pattern. An entropy generation model can be established based on the detailed flow pattern to study the irreversibility of the flow boiling in microgravity field. Since the descriptive boiling models (RPI [32], LEE [33]) are also based on empirical coefficients, few studies used these phase–change models for the analysis of entropy generation in phase change processes. In order to remove the influence of empirical coefficients, this paper employs a phase–change model proposed by Sun [34] to perform accurate simulations of the phase–change process in order to obtain universal properties of the entropy generation law in a microgravity field.

Considering the value of analysis based on the second law of thermodynamics in the optimization of heat transfer systems, the target of this essay is to discuss the entropy generation characteristics of the flow boiling inside a tunnel in microgravity through numerical simulation, and to prepare for the thermodynamic optimizations of boiling heat exchange equipment in the microgravity field. A new entropy generation model based on the flow pattern and the phase–change process is developed in this study. The simulations are performed on ANSYS-FLUENT. The relationship between the entropy generation and the heat flux, as well as the velocity, is taken into consideration to obtain a deeper understanding of the irreversibility of flow boiling in microgravity. An evaluation number *E*_P_, which is a criterion combining the first law and the second law of the thermodynamics, is introduced in this paper to evaluate the performance of the boiling process.

## 2. Mathematical Model

### 2.1. Governing Equations of CFD Calculation

The volume of fluid (VOF) model is employed in this paper to simulate the liquid-vapor flow of the flow boiling in microgravity field. The ability of VOF model to capture the interface between vapor and liquid is achieved by introducing the volume fraction $\alpha_{l}$ [33]. If $\alpha_{l}$ stands for the volume fraction of liquid and the $\alpha_{v}$ stands for the volume fraction of vapor, the following situations are possible:

$\alpha_{l}\;$= 1: The cell is occupied by liquid.

$\alpha_{v}$ = 1: The cell is occupied by vapor.

0 <$\;\alpha_{v}\;$< 1: The interface between liquid and vapor exists in the cell. 

**Continuity Equation**

(1) $$\frac{\partial \rho_{l}\alpha_{l}}{\partial t}+\nabla \cdot \left(\rho_{l}\alpha_{l}V\right)={\overset{•}{m}}_{vl}-{\overset{•}{m}}_{lv}$$

**Momentum Equation**

(2) $$\frac{\partial }{\partial t}\left(\rho u\right)+\bar{V}\cdot \nabla \left(\rho u_{i}\right)=\mu \nabla^{2}u_{i}-\frac{\partial p}{\partial x}+F_{stx}$$

(3) $$\frac{\partial }{\partial t}\left(\rho v\right)+\bar{V}\cdot \nabla \left(\rho u_{j}\right)=\mu \nabla^{2}u_{j}-\frac{\partial p}{\partial y}+F_{sty}+G$$

Here, *u* and *v* are the velocity components, p represents the pressure. ρ stands for the density of the mixture phase and μ stands for the viscosity. ρ and μ can be acquired by the following equations.

(4) $$\rho =\alpha_{l}\rho_{l}+\alpha_{v}\rho_{v}$$

(5) $$\mu =\alpha_{l}\mu_{l}+\alpha_{v}\mu_{v}$$

**Energy Equation**

(6) $$\frac{\partial }{\partial t}\left(\rho E\right)+\bar{V}\cdot \nabla \left(\rho E+p\right)=\nabla \cdot \left(\kappa_{eff}\nabla T\right)+S_{T}+S_{B}$$

Here, $S_{B}$ is the energy source term, which is used to calculate the heat transfer coupled with the mass transfer in the boiling phenomenon. $S_{T}\;$represents the energy source term produced by viscosity of the fluid. The summary of $S_{T}$ and $S_{B}$ can be defined as $S_{E}$.

The phase–change model proposed by Sun is adopted in this paper to simulate the phase–change process in the boiling phenomenon [34]. The governing equations of the phase–change model are shown as the following.

**Evaporation**

(7) $$\mathrm{Mass}\;\mathrm{description}:\;\;\;\;\overset{•}{m_{lv}}=\overset{•}{m_{vl}}=\frac{Q_{C_{I},in}}{h_{fg}V_{C_{Is}}}+\frac{Q_{C_{Is},in}}{h_{fg}V_{C_{Is}}}$$

(8) $$\mathrm{Energy}\;\mathrm{description}:\;\;\;\;\;S_{B}=\frac{-Q_{C_{I}s,in}}{V_{C_{I}}}$$

**Condensation**

(9) $$\mathrm{Mass}\;\mathrm{description}:\;\;\;\overset{•}{m_{vl}}=-\overset{•}{m_{lv}}=\frac{Q_{C_{I},out}}{h_{fg}V_{C_{Is}}}+\frac{Q_{C_{Is},out}}{h_{fg}V_{C_{Is}}}\;\;\;\;\;$$

(10) $$\mathrm{Energy}\;\mathrm{description}:\;\;\;S_{B}=\frac{-Q_{C_{Is},out}}{V_{C_{I}}}$$

For details and the validation of the phase–change model developed by Sun, the reader is referred to the essay [34].

### 2.2. Simulation Model

**Figure 1:**
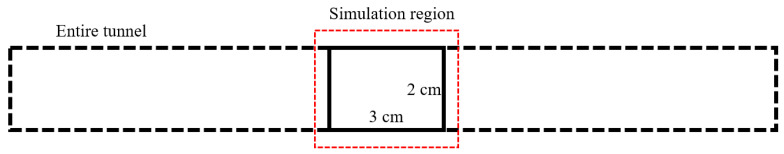
The schematic diagram of the geometry model.

The geometry model established in this paper is displayed in Figure 1.

The periodic boundary condition is adopted in this paper to simulate the boiling process to minimize the negative influence caused by boundary conditions. The employment of the periodic boundary conditions, in fact, forms a longer tunnel during the processing of the simulation. The length of the flowing distance can be obtained by the following equation:

(11) L=n×l

Here, *L* stands for the length of the flow distance, n is the number of fluid domains that the fluid flows through, and *l* is the length of the fluid domain. The upside boundary of the domain is set as a heat insulation wall, whereas the bottom of the domain is set as a hot wall. The insulation–heating wall strategy highlights the difference between the behavioral characteristics of the bubbles in the fluid domain, those under the gravity field, and those under microgravity field. The heat flux from the wall ranges from 10,000 W/m^2^ to 50,000 W/m^2^. The impact of heat flux on entropy production characteristics in the flow boiling is studied every 10,000 W/m^2^. A set of velocities, ranging from 1 m/s to 4 m/s every 1 m/s, is taken into consideration to observe the tendency of the entropy generation in flow boiling with different flow patterns. The velocity acceleration in the y direction is set as −0.0005 m/s^2^. The water vapor is employed as the primary phase whereas the liquid water is employed as the secondary phase. The transient simulation is adopted in this paper with the courant number smaller than 0.25 to ensure a set of more accurate results.

**Figure 2:**
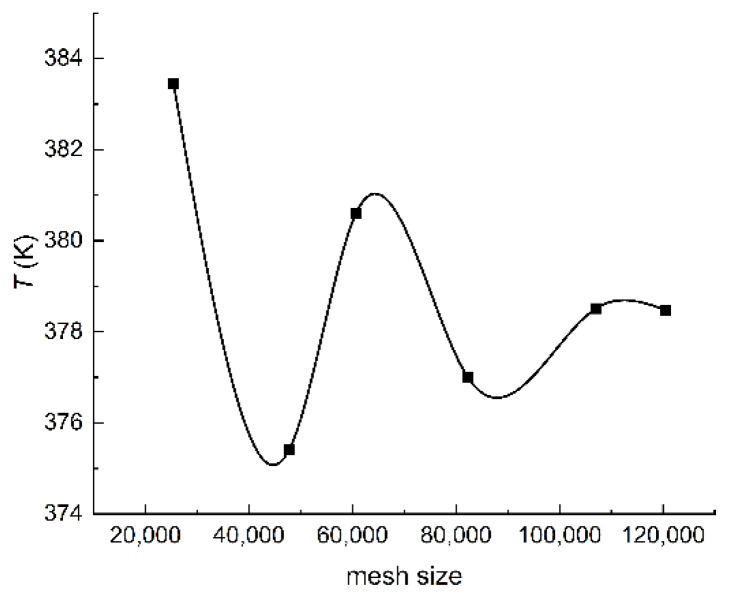
The temperature of the hot wall vs. mesh size.

Five meshes are taken into consideration to check the grid influence on the simulation results for a given working condition. The velocity in the simulations is 1 m/s and the heat flux in the simulations is 10,000 W/m^2^. The time-average temperatures of the hot wall from 0 s to 0.05 s is chosen as the criteria. The results obtained in the simulations are displayed in Figure 2.

It can be observed from Figure 2 that the temperature of the hot wall tends to stabilize when the mesh size comes up to 107,067. The mesh with 107,067 cells is selected in this paper to perform accurate simulations at a low numerical computational cost. 

## 3. Entropy Generation Model

The transport equation for entropy generation in Cartesian coordinates can be obtained based on the second law of thermodynamics as the following [35]:

(12) $$\frac{\partial e_{m}}{\partial t}\left(\rho s\right)+\frac{\partial }{\partial x_{i}}\left(\rho_{m}s_{m}u_{i,m}\right)=\frac{\partial J_{i}^{s}}{\partial x_{i}}+{\dot{S}}_{gen}^{\prime \prime \prime }$$

Here, $J_{i}^{s}$ is the entropy flux in the *i*^th^ orientation, and the $S_{gen}^{\prime \prime \prime }$ is the entropy generation rate. The governing entropy equation is considered to be able to deduce the entropy generation model from the simulation region as follows [35]:

(13) $$\rho_{m}\left(\frac{\partial s_{m}}{\partial t}+u_{i,m}\frac{\partial s_{m}}{\partial x_{i}}\right)=\frac{\partial J_{i}^{s}}{\partial x_{i}}+\frac{\Phi }{T_{m}}+\frac{\Phi_{pc}}{T_{m}}+\frac{\Phi_{\Theta }}{T_{m}^{2}}$$

Here, $\frac{\Phi }{T_{m}}$ is the entropy generation produced by the viscosity, $\frac{\Phi_{pc}}{T_{m}}$ is the entropy generation produced by the phase–change, and $\frac{\Phi_{\Theta }}{T_{m}^{2}}$ is the entropy generation produced by heat transfer. The variables in the equations solved in the RANS approach can be sorted into a time-average part and a fluctuating part as follows:

$$s_{m}=\bar{s_{m}}+s_{m}^{\prime },\;T_{m}=\bar{T_{m}}+T_{m}^{\prime },\;u_{i,m}=\bar{\;u_{i,m}}+\;u_{i,m}^{\prime }$$

Here, *J* represents the mass transfer rate. The terms in Equation (13) are replaced with the time-averaged variables as follows. 

**Convective Terms**

The time-averaged entropy generation source term caused by convection at the left side of Equation (13) is shown as [35]:

(14) $$\bar{\frac{\partial s_{m}}{\partial t}+u_{i,m}\frac{\partial s_{m}}{\partial x_{i}}}=\frac{\partial \bar{s_{m}}}{\partial t}+\bar{u_{i,m}}\frac{\partial \bar{s_{m}}}{\partial x_{i}}+\frac{\partial \bar{u_{i,m}^{\prime }s_{m}^{\prime }}}{\partial x_{i}}\;\;$$

**Entropy Generation by Dissipation**

The time-averaged entropy generation caused by viscous dissipation consists of two terms: the time-averaged flow section and the fluctuating flow section, which is shown as follows [35]:

(15) $$\bar{\frac{\Phi }{T_{m}}}=\frac{\mu_{m}}{\bar{T_{m}}}\left\{2{\left(\frac{\partial \bar{u_{i,m}}}{\partial x_{i}}\right)}^{2}+{\left(\frac{\partial \bar{u_{i,m}}}{\partial x_{j}}+\frac{\partial \bar{u_{j,m}}}{\partial x_{i}}\right)}^{2}\right\}+\frac{\mu_{m}}{\bar{T_{m}}}\left\{2{\left(\frac{\partial \bar{u_{i,m}^{\prime }}}{\partial x_{i}}\right)}^{2}+{\left(\frac{\partial \bar{u_{i,m}^{\prime }}}{\partial x_{j}}+\frac{\partial \bar{u_{j,m}^{\prime }}}{\partial x_{i}}\right)}^{2}\right\}$$

Here, the $T_{m}^{\prime }$ is neglected.

**Entropy Generation by Heat Transfer**

The time-averaged entropy generation due to the heat transfer consists of two terms: the time-averaged temperature gradient section and the fluctuating temperature gradient section, which is shown as follows:

(16) $$\bar{\frac{\Phi_{\Theta }}{T_{m}^{2}}}=\frac{\kappa_{m}}{\bar{T_{m}^{2}}}\left\{{\left(\frac{\partial \bar{T_{m}}}{\partial x_{i}}\right)}^{2}\right\}+\frac{\kappa_{m}}{\bar{T_{m}^{2}}}\left\{{\left(\frac{\partial \bar{T_{m}^{\prime }}}{\partial x_{i}}\right)}^{2}\right\}$$

**Entropy Generation by Phase Change**

The time-averaged entropy generation caused by the phase change is shown as:

(17) $$\bar{\frac{\Phi_{pc}}{T_{m}}}=\frac{h_{fg}\cdot \bar{J}}{\bar{T_{m}}}$$

The transport equation of the average entropy in this paper can be obtained by substituting the time-averaged terms into Equation (13).

(18) $$\rho_{m}\left(\frac{\partial s_{m}}{\partial t}+u_{i,m}\frac{\partial s_{m}}{\partial x_{i}}\right)=\frac{\partial J_{i}^{s}}{\partial x_{i}}-\rho_{m}\frac{\partial \bar{u_{i,m}^{\prime }s_{m}^{\prime }}}{\partial x_{i}}+\frac{\mu_{m}}{\bar{T_{m}}}\left\{2{\left(\frac{\partial \bar{u_{i,m}}}{\partial x_{i}}\right)}^{2}+{\left(\frac{\partial \bar{u_{i,m}}}{\partial x_{j}}+\frac{\partial \bar{u_{j,m}}}{\partial x_{i}}\right)}^{2}\right\}+\frac{\mu_{m}}{\bar{T_{m}}}\;\left\{2{\left(\frac{\partial \bar{u_{i,m}^{\prime }}}{\partial x_{i}}\right)}^{2}+{\left(\frac{\partial \bar{u_{i,m}^{\prime }}}{\partial x_{j}}+\frac{\partial \bar{u_{j,m}^{\prime }}}{\partial x_{i}}\right)}^{2}\right\}+\frac{\kappa_{m}}{\bar{T_{m}^{2}}}\left\{{\left(\frac{\partial \bar{T_{m}}}{\partial x_{i}}\right)}^{2}\right\}+\frac{\kappa_{m}}{\bar{T_{m}^{2}}}\left\{{\left(\frac{\partial \bar{T_{m}^{\prime }}}{\partial x_{i}}\right)}^{2}\right\}+\frac{h_{fg}\cdot \bar{J}}{\bar{T_{m}}}$$

The existence of the velocity and temperature fluctuations results in a set of unclosed equations. The unclosed entropy source terms can be replaced through the analysis of the *k*-*ε* turbulence model, which is shown as follows [35].

(1) The entropy generation caused by turbulent dissipation can be expressed as:

(19) $${\dot{S}}_{gen,D}^{\prime \prime \prime }=\frac{\rho_{m}\varepsilon }{\bar{T_{m}}}$$

(2) Utilizing the Boussinesque-like approach [36], the entropy generation caused by fluctuating temperature gradients is described as follows:

(20) $${\dot{S}}_{gen,C^{\prime }}^{\prime \prime \prime }=\frac{a_{t}}{a}\frac{\kappa_{m}}{\bar{T_{m}^{2}}}\cdot {\left(\frac{\partial \bar{T_{m}}}{\partial x_{i}}\right)}^{2}$$

The entropy generation caused by the average temperature gradients and that caused by the fluctuating temperature gradients can be combined as follows:

(21) $${\dot{S}}_{gen,C^{\;}}^{\prime \prime \prime }=\frac{\kappa +\kappa_{t}}{\bar{T_{m}^{2}}}\cdot {\left(\frac{\partial \bar{T_{m}}}{\partial x_{i}}\right)}^{2}=\frac{\kappa_{eff}}{\bar{T_{m}^{2}}}\cdot {\left(\frac{\partial \bar{T_{m}}}{\partial x_{i}}\right)}^{2}$$

The entropy generation model in the boiling process can be concluded as follows:

(22) $${\dot{S}}_{gen}^{\prime \prime \prime }=\underset{heat\;transfer}{\underbrace{\frac{\kappa_{eff}}{T^{2}}{\left(\nabla T\right)}^{2}}}+\underset{viscous\;dissipation}{\underbrace{\frac{\mu_{m}}{T_{m}}\left\{2{\left(\frac{\partial \bar{u_{i,m}}}{\partial x_{i}}\right)}^{2}+{\left(\frac{\partial \bar{u_{i,m}}}{\partial x_{j}}+\frac{\partial \bar{u_{j,m}}}{\partial x_{i}}\right)}^{2}\right\}}}+\underset{\begin{array}{c} turbulent\\ dissipation \end{array}}{\underbrace{\frac{\rho_{m}\varepsilon }{T_{m}}}}+\underset{phase-change}{\underbrace{\frac{h_{fg}\cdot J}{T}}}$$

Here, $\kappa_{eff}$ stands for the equivalent heat conductivity coefficient, which can be calculated as:

(23) $$\kappa_{eff}=\alpha_{l}{\left(\kappa_{q}+\kappa_{t}\right)}_{l}{}_{\;}+\alpha_{v}{\left(\kappa_{q}+\kappa_{t}\right)}_{v}{}_{\;}$$

Here, $\kappa_{q}$ represents thermal conductivity and the $\kappa_{t}$ represents the turbulence closure model. The product of J and the latent heat of vaporization, which is the total heat flux participating in the boiling process, can be calculated as follows:

(24) $$q_{total}=J\cdot h_{fg}$$

$q_{total}$ can be gained through the loops of the cells inside the fluid domain during the simulation. The total entropy generation can be obtained through:

(25) $${\dot{S}}_{gen}^{\;}=\int_{V}^{\;}{\dot{S}}_{gen}^{\prime \prime \prime }dV$$

**Figure 3:**
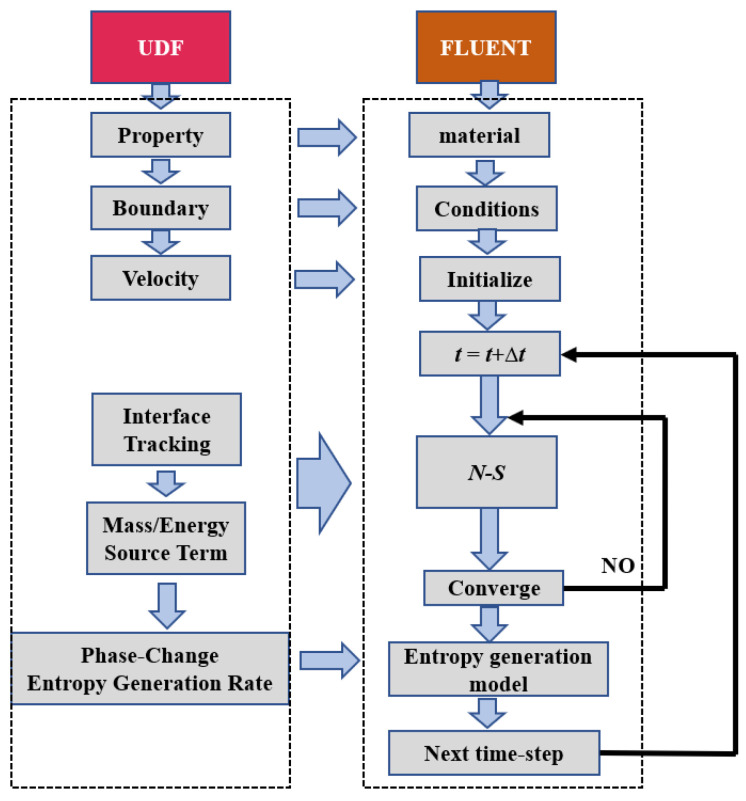
The technical method.

It is noticeable that the heat conduction and the viscosity dissipation exist in the whole domain, whereas the condensation and evaporation do not; thus, it is necessary to classify the cells in the domain to calculate the phase–change entropy generation and other entropy generations. The cells in the domain can be sorted into two categories: (1) the cells full with water (vapor) where the boiling cannot occur; (2) the cells with the interface in them and the cells full with saturated water where the boiling happens. The category of the local cell needs to be updated every timestep with the growth of the bubbles when the simulation proceeds. The technique solution adopted in this paper is shown in Figure 3.

## 4. Results and Discussion

### 4.1. Validation of the Simulation Model

The boiling phase–change model employed in this paper removes the influence of empirical coefficients and directly simulates the boiling behavior based on bubble dynamics. In order to verify the numerical model established in this paper, a set of the simulations are taken into consideration with the results obtained in the subcooled flow boiling experiments conducted by Zhang on NASA’s KC-135 aircraft [36]. The height of the flow channel is 0.5 cm, and the coolant used by Zhang is FC-72. Due to the different working medium of the experiments and numerical simulations, a comparative analysis is needed for the flow field characteristics. The Reynolds number of the bubbles is defined as follows [37].

(26) $$Re_{b}=\frac{\rho \left(v_{l}-v_{b}\right)l}{\mu }$$

The bubble-liquid drag force coefficient is defined as [37]:

(27) $$C_{D}=0.5191-\frac{1662.5}{Re_{b}}+\frac{5,416,700}{Re_{b}{}^{2}}(Re_{b}\;>\;10,000)$$

The working conditions and the parameters of the flow field characteristics of the experiment and numerical simulation are shown in the Table 1.

**Figure 4:**
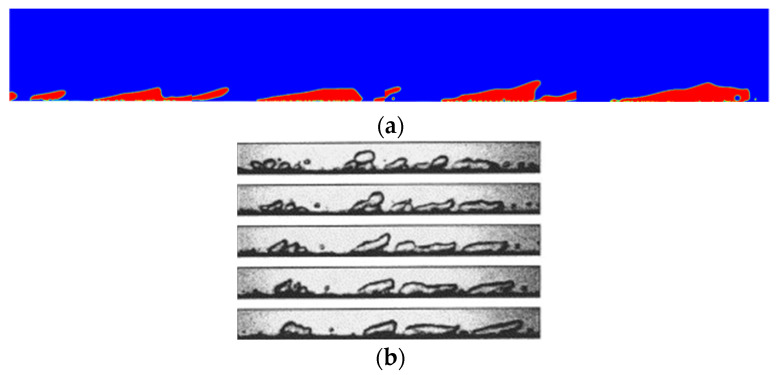
The comparison between the bubbles obtained in simulations and the experiments. (**a**) Bubbles obtained in simulation. (**b**) Bubbles obtained in experiments. Reprinted with permission from Ref. [36]. Copyright 2005, Elsevier.

It can be seen from the bubble-liquid drag force coefficients that the two-phase flow field characteristics of the numerical model are similar to those of the experiments, which means that the bubble distribution of both can be compared and analyzed. The two-phase distribution of nucleate boiling obtained from the experiment is compared with the two-phase distribution of nucleate boiling obtained from the simulation, as shown in Figure 4:

The behavior of the bubbles in nucleate boiling that were obtained in the simulation are found to be consistent with the behavior of the bubbles observed in the experiment. The bubbles are absorbed on the heated walls and deformed toward the mainstream flow direction. The agreement of the bubbles’ behavior between experiments and numerical simulation results indicates that the model in this paper can predict the boiling behavior in a microgravity field within an acceptable degree of uncertainty.

### 4.2. Influence of the Heat Flux

**Figure 5:**
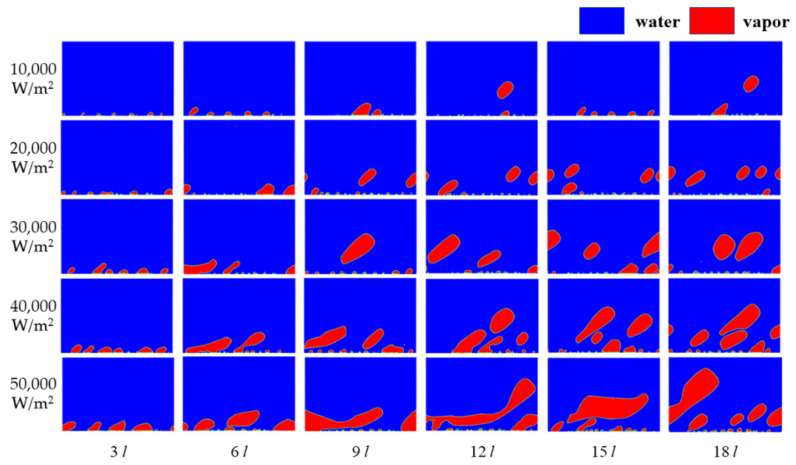
The distribution of water vapor when the velocity is 1 m/s.

The characteristics of the local entropy generation in the boiling process are significantly affected by the distribution of the liquid phase and the vapor phase, which are different from the single-phase flow. The distribution of the liquid phase and the vapor phase is determined by the boiling status. It is necessary to guarantee the consistency of the boiling status when discussing the influence of the heat flux on local entropy generation. The phase distribution with a different heat flux is presented in Figure 5 when the velocity is 1 m/s.

**Figure 6:**
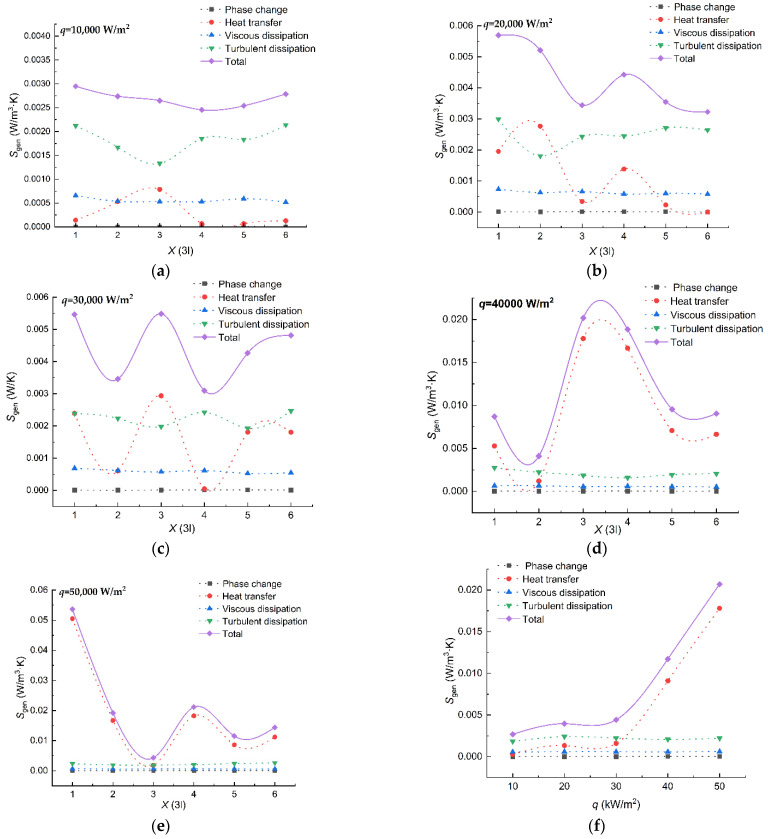
Distribution of the local entropy generation with different heating distance when the velocity is 1 m/s; (**a**) *q* = 10,000 W/m^2^; (**b**) *q* = 20,000 W/m^2^; (**c**) *q* = 30,000 W/m^2^; (**d**) *q* = 40,000 W/m^2^; (**e**) *q* = 50,000 W/m^2^; (**f**) The tendency of the average entropy generation of the entire tunnel.

The blue region in Figure 5 stands for water, whereas the red region stands for vapor. It can be observed that every boiling status involves nucleate boiling under the working conditions shown. The entropy generation produced by the heat transfer, viscous dissipation, turbulent dissipation, and the phase–change with a different heat flux is presented at a velocity of 1 m/s in Figure 6.

It can be observed from Figure 6a–e that different kinds of entropy generation feature different tendencies. Fluctuations of heat transfer entropy generation and turbulent dissipation entropy generation along the heating distance can be obtained in Figure 6a–e. The fluctuation range of the heat transfer entropy generation has a positive correlation with the heat flux. The peak of the heat transfer entropy generation is 0.000785 W/m^3^·K when the heat flux is 10,000 W/m^2^, whereas the peak of the heat transfer entropy generation is 0.0505 W/m^3^·K when the heat flux is 50,000 W/m^2^. It can be seen that the peak of the heat transfer entropy differs by two orders of magnitude under the above two conditions; however, the heat flux has no influence on the bottom of the heat transfer entropy generation curves. The bottom values of the heat transfer entropy generation under all conditions are all of the order of 10^−5^. The fluctuations of turbulent dissipation entropy generation seem to have no correlation with the heat flux, which are different from those of the heat transfer entropy generation. It can be concluded from Figure 6f that the tendencies of the phase–change entropy generation and the viscous dissipation entropy generation are not affected by the heating distance. The difference between the two entropy generations is the influence of heat flux. The heat flux has influence on the phase change entropy generation, but not on the viscous dissipation entropy generation. The phase–change entropy generation increases from 0.000004 W/m^3^·K to 0.000012 W/m^3^·K when the heat flux increases from 10,000 W/m^2^ to 50,000 W/m^2^. The total entropy generation tends to be proportional to the heat flux with the contributions of all kinds of entropy generation.

**Figure 7:**
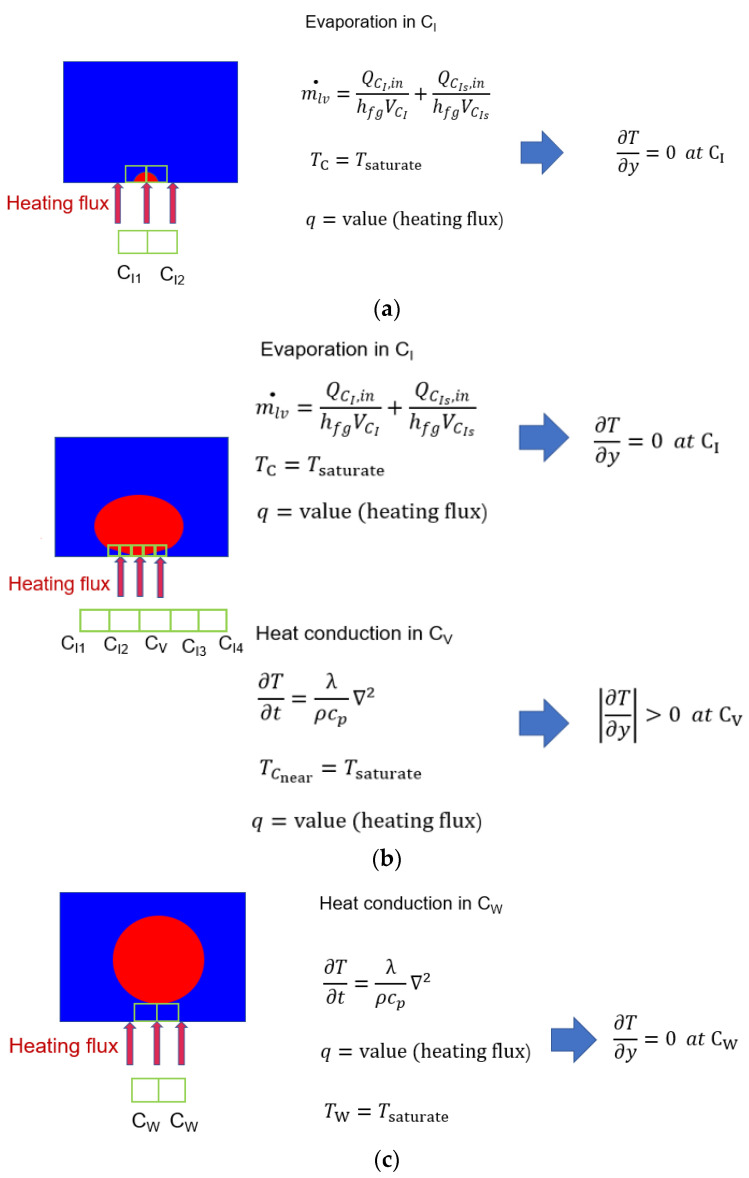
The behavior of bubbles. (**a**) The bubble at the induced status. (**b**) The bubble at the growing status. (**c**) The exfoliated bubble.

The temperature gradient in the bubbles increases with the increase in heat flux, which contributes to the phenomenon of heat transfer entropy generation presented in Figure 6f. The behavior features of the bubbles is shown in Figure 7.

The saturated water, the temperature of which is 373.15 K, absorbs heat near the hot wall, generating a bubble under a constant temperature. The condition of constant temperature indicates that the temperature gradient in this region is equal to zero, where no heat transfer entropy generation exists. The phenomenon and the descriptive equations in this state are presented in Figure 7a. A situation with multiple bubbles has little difference. The value of the heat transfer entropy generation does not equal 0 in the bottom of the heat transfer entropy generation curves in Figure 6a–e. It is caused by the inconsistent behaviors of the bubbles in a relatively large region. Most of the bubbles are at the first stage of the growth cycle, whereas a few of the bubbles are at the next stage of the growth cycle. The second stage of the bubble growth cycle is the process in which the bubbles grow larger. The region near the hot wall in this process can be sorted into two categories based on the phase distribution. One category of regions contains the interface between water and vapor, where the evaporation occurs. The features of the temperature field in this kind of region share the same characteristics with that in the first stage of the bubble growth cycle. The other category of regions in the second stage of the bubble growth cycle is only filled with vapor, where the heat conduction occurs. A rise in temperature can be observed in this region for the vapor absorption of the heat from the hot wall. As a result, the temperature gradient of the vapor region gradually increases, as does the heat transfer entropy generation. The phenomenon and the descriptive equations are presented in Figure 7b. When the volume of the bubbles achieves a certain level, the main flow will bring the bubbles away from the hot wall. The hot wall will be cooled down, preparing for the next period of the bubble growth. The phenomenon and the descriptive equations are presented in Figure 7c. The combination of the periods contributes to the undulation of the heat transfer entropy generation in Figure 6.

The dimensionless Bejan number (*Be*) [35] is introduced to make a comparison between the contributions of the heat transfer entropy generation make to the total entropy under different heat flux with different velocity:

(28) $$Be=\frac{{\dot{S}}_{\mathrm{gen},C}}{{\dot{S}}_{\mathrm{gen}}}$$

**Figure 8:**
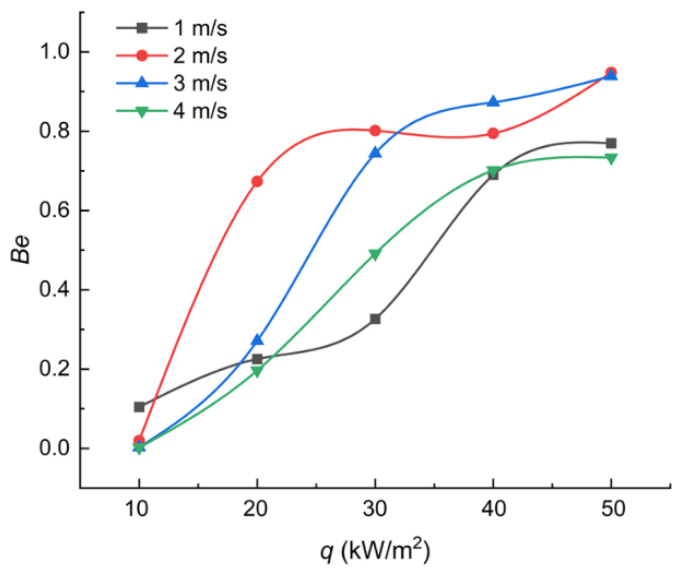
Trends of the *Be* number.

The *Be* numbers under different working conditions are presented in Figure 8.

It can be seen in Figure 8 that the *Be* number has a positive relationship with the heat flux under all the working conditions. The tendency of the *Be* reveals that the heat transfer entropy generation becomes the dominant factor of the total entropy generation with the increase in heat flux. The process of the heat transfer entropy generation becoming the dominant factor, indicates that the distribution of the vapor generated during boiling determines the reversibility in the whole tunnel when the heat flux increases. It is necessary to take the distribution of the phase into consideration when a thermal system adopting boiling is optimized.

### 4.3. Influence of the Velocity

**Figure 9:**
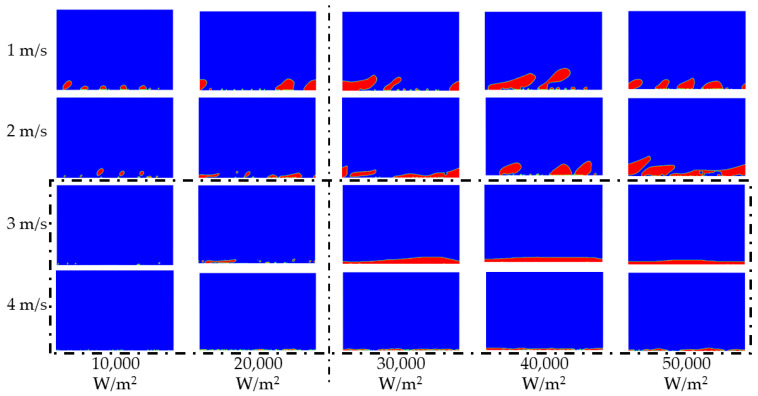
The water vapor distribution.

The flow boiling status inside a tunnel in a gravity field is mainly affected by the heat flux. The flow boiling status inside a tunnel in microgravity field is more affected by the working fluid velocity, which is different from that in the gravity field. The two-phase distribution at different velocities is shown in Figure 9.

Nucleate boiling can be found in the tunnel with all the heat fluxes from Figure 9 when the velocity is 1 m/s. A similar phenomenon in boiling status can be observed in Figure 9 when the velocity is 2 m/s compared with when the velocity is 1 m/s. The difference between the bubbles at the velocities of 1 m/s and 2 m/s is the size of the bubbles and the deformation of the bubbles caused by the main flow velocity. A set of smaller bubbles can be gained in all working conditions when the velocity is 2 m/s. The tendency of the water vapor distribution to diverge when the heat flux differs, occurs when the velocity is 3 m/s. Nucleate boiling still can be observed at working conditions when the heat flux is 10,000 W/m^2^ and 20,000 W/m^2^, respectively; however, nucleate boiling translates to film boiling when the heat flux is 30,000 W/m^2^, 40,000 W/m^2^, and 50,000 W/m^2^, respectively. This tendency concerning water vapor distribution diverging when the heat flux differs, still exists when the velocity is the 4 m/s. Few bubbles can be found near the hot wall when the heat flux is 10,000 W/m^2^ and 20,000 W/m^2^, respectively. A vapor film that doesn’t cover the entire hot wall can be observed when the heat flux is 30,000 W/m^2^, 40,000 W/m^2^, and 50,000 W/m^2^, respectively. A common rule is shared amongst the working conditions, that the size of bubbles (films) tend to be smaller with the increase in velocity. The distribution of the water vapor phase determines the characteristics of irreversibility. The dimensionless entropy generation number is adopted to take a comparison between the characteristics of the local irreversibility under a different heat flux. The entropy generation number is defined as follows.

(29) $$N_{s}=\frac{{\dot{S}}_{\mathrm{gen}}T_{b}}{Q}$$

**Figure 10:**
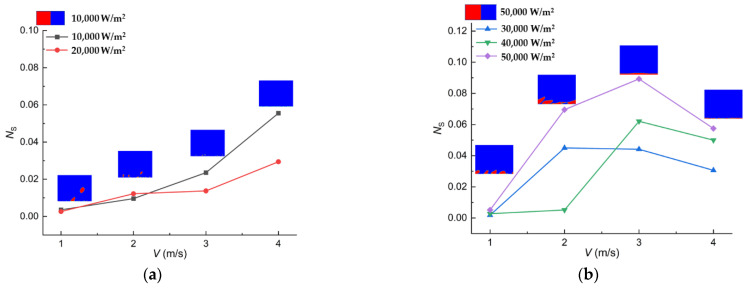
The entropy generation number vs. velocity. (**a**) *N_S_* vs. velocity when *q* is 10,000 W/m^2^ and 20,000 W/m^2^. (**b**) *N_S_* vs. velocity when *q* is 30,000 W/m^2^, 40,000 W/m^2^ and 50,000 W/m^2^.

Here, ${\dot{S}}_{\mathrm{gen}}$ is the total entropy generation in the fluid domain, *T*_b_ stands for the bulk temperature, and Q represents the heat input to the domain. The entropy generation numbers under a different heat flux are presented in Figure 10, as are a set of representative water vapor distributions, respectively.

It can be observed from Figure 10a that irreversibility has a positive correlation with the velocity when the heat flux is 10,000 W/m^2^ and 20,000 W/m^2^. The irreversibility at working conditions with high heat fluxes tends to increase then decrease as shown in Figure 10b, which is totally different from the case with low heat flux.

The irreversibility characteristics gained in Figure 10 are caused by the transition of the boiling status. The transition of the boiling status, which is different from that resulted from the heat input in the gravity field, results from the velocity. It is known that the velocity of the bulk flow is much higher than that of the fluid layer near the wall, where the velocity nearly equals 0. The shear stress between different flow layers with different velocities moves the bubbles generated near the hot wall away. The effect of the shear stress on the direction of the bubble motion is shown along the direction of the fluid, which is completely different from the effect of the gravity. The higher velocity, the more pronounced the tendency of the bubble motion. The small bubbles generated at the working conditions of 10,000 W/m^2^ and 20,000 W/m^2^ do not interact with each other, whereas the bigger bubbles tend to emerge near each other at the working conditions of higher heat fluxes. The vapor film formed by the merging between the bubbles actually leads to a high temperature gradient in the domain, contributing to a higher irreversibility in the fluid region.

### 4.4. Performance Evaluation

The operating condition with the lowest irreversibility can be obtained by the employment of the entropy generation number analysis; however, the operating condition with the lowest irreversibility does not promise an optimal heat transfer performance of the tunnel in microgravity field. Therefore, the evaluation number *E*_P_, which takes the first and the second law of the thermodynamics into consideration, is described as:

(30) $$E_{P}=\frac{Ja}{N_{S}}$$

**Figure 11:**
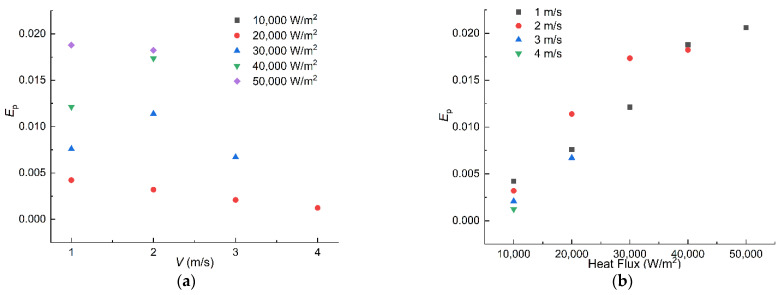
Performance of the boiling process with different working conditions. (**a**) *E*_P_ vs. Velocity. (**b**) *E*_P_ vs. heat flux.

Here, Ja is the Jakob number which characterizes the heat transfer ability of nucleate boiling, and $N_{S}$ is the entropy generation number mentioned above; therefore, the evaluation number *E*_P_ can characterize the boiling heat transfer capacity per unit of entropy generation. The *E*_P_ criterion is adopted to analyze the influence of velocity and heat flux on the performance of the boiling phenomenon in the tunnel. It is noticeable that not all the operating conditions in Figure 9 are taken into the analysis for the occurrence of the film boiling in some working conditions. The occurrence of the film boiling may lead to the melting of the hot wall, which is unacceptable in industrial facilities. The performances of the flow boiling in the tunnel are presented in Figure 11.

It can be observed from Figure 11a that the number of meaningful operating conditions tends to decrease with the increase in velocity. It can be seen in Figure 11b that the $E_{P}$ has a positive correlation with the heat flux when the boiling status remains at nucleate boiling. The heat flux at the optimal operating condition with a certain velocity is the highest heat flux. The positive correlation between $E_{P}$ and the heat flux indicates that the negative impact of the irreversibility increase on the tunnel is compensated by the enhancement of the boiling heat transfer capacity when the heat flux rises. In fact, the heat flux at the optimal operating condition is exactly the same as the critical heat flux (CHF) at certain working conditions. The agreement of the optimal heat flux obtained by the evaluation number *E*_P_ and the CHF shows the rationality of the criterion developed in this paper.

## 5. Conclusions

An entropy generation analysis of flow boiling heat transfer in the microgravity field is conducted in this paper. The phase–change model developed by Sun is employed to simulate the evaporation–condensation during the flow boiling process. The entropy generation model is established in accordance with the second law of thermodynamics. The velocity and the heat flux are taken into consideration to figure out their influence on the entropy generation characteristics. Moreover, an evaluation number is introduced in this paper to investigate the performance of the flow boiling in the tunnel. The conclusions are drawn as follows:

(1) The local distribution of the water vapor has a great influence on local entropy generation. A high local entropy generation will be obtained only when heat conduction in vapor occurs near the hot wall, whereas a low local entropy generation will be obtained when heat conduction in water or evaporation occurs near the hot wall. The vapor–liquid distribution near the heating wall changes alternately as the bubbles grow and fall off in nucleate boiling, causing the total entropy generation to fluctuate with the increase of the heating distance. The near-wall region is filled with vapor in film boiling, which causes the total entropy generation to rise continuously with the increase of the heating distance.
(2) The heat transfer entropy generation becomes the major contributor of the total entropy generation with the increase of the heat flux. Unlike the heat transfer entropy generation, the turbulent dissipation entropy generation and the viscous dissipation entropy generation are only influenced by the velocity of the flow. The phase–change entropy generation is more complex and is determined by the boiling state. The boiling state is under the coupled influence of velocity and heat flux. As the result, it is difficult to analyze the effect of a single variable on phase–change entropy generation.
(3) The velocity in the tunnel has a great effect on the boiling status and determines the entropy generation in the tunnel. The increase of the velocity at a low heat flux will restrain the nucleate boiling, reducing the irreversibility in the tunnel; however, the increase of the velocity at a high flux will promote the boiling status transition from nucleate boiling to film boiling, creating more irreversibility in the tunnel.
(4) The optimal operating condition can be achieved through the introduction of the evaluation number *E*_P._ A positive correlation between the heat flux and the *E*_P_ can be observed when the velocity keeps constant. As a result, the CHF is the optimal choice under the first law and the second law of the thermodynamics.

In general, a numerical simulation-based entropy generation model is developed to investigate the irreversibility of the boiling flow in the microgravity field. A phase–change model based on Fourier conductivity is employed in this paper to simulate the phase change process and to establish the flow boiling entropy generation model. A deeper understanding of the boiling process, including the boiling state and the thermodynamic irreversibility in the microgravity field, can be obtained based on the mathematical model and the entropy yield analysis model developed in this paper, which is desirable to make contributions when a thermodynamic facility involved the boiling flow process in microgravity field is designed.

## Figures and Tables

**Table 1:** The experiments vs. the numerical model.

	Simulation	Experiment [33]
medium	water	FC 72
$\rho_{l}$ (kg/m^3^)	998	1680
*V* (m/s)	1	0.14
μ (kg/(m·s))	0.001003	0.00064
*L* (m)	0.02	0.005
$C_{D}$	5,416,700.436	5,416,700.012

## Data Availability

Not applicable.

## Nomenclature

$\alpha_{l}$	Volume fraction of liquid
$\alpha_{v}$	Volume fraction of vapor
*Be*	Bejan number
$C_{D}$	Bubbles-liquid drag force coefficient
$h_{fg}$	Latent heat (kJ/kg)
J	Flow
Ja	Jakob number
$J_{i}^{s}$	Flow
*l*	Length of the fluid domain (cm)
${\overset{•}{m}}_{vl}$	Condensation mass (kg/m^3^)
${\overset{•}{m}}_{lv}$	Evaporation mass (kg/m^3^)
$N_{s}$	Entropy generation number
p	Pressure (Pa)
$Q_{C_{Is},in}$	Heat into the interface cell (J)
$Q_{C_{Is},out}$	Heat out the interface cell (J)
$Re_{b}$	Reynolds number of the bubbles
$S_{\;}$	Source term
$S_{T}$	Energy source term generated by viscosity (W/m^3^)
$S_{B}$	Energy source term generated by boiling (W/m^3^)
${\dot{S}}_{gen}^{\prime \prime \prime }$	Entropy generation rate (J/(K·s·m^3^))
${\dot{S}}_{gen}^{\;}$	Entropy generation (J/(K·m^3^))
$s_{m}$	Entropy variable (J/(kg·K)
$\bar{s_{m}}$	Time-averaged entropy variable (J/(kg·K)
$s_{m}^{\prime }$	Fluctuating entropy variable (J/(kg·K)
T	Temperature (K)
$\bar{T_{m}}$	Time-averaged Temperature (K)
$T_{m}^{\prime }$	Fluctuating Temperature (K)
$u_{i}$	x components of velocity (m/s)
$u_{j}$	y components of velocity (m/s)
$\bar{u_{i}}$	Time-averaged x components of velocity (m/s)
$\bar{u_{j}}$	Time-averaged y components of velocity (m/s)
$u_{i}^{\prime }$	Fluctuating x components of velocity (m/s)
$u_{j}^{\prime }$	Fluctuating y components of velocity (m/s)
*VOF*	Volume of fluid
*Greek symbols*	
ρ	Density (kg/m^3^)
μ	Viscosity (kg/(m·s))
ε	Turbulent dissipation rate
$\kappa_{\;}$	Thermal conductivity (W/(m·K))
$\kappa_{eff}$	Effective thermal conductivity (W/(m·K))
*Subscript*	
${}^{\prime }$	Fluctuating
$\bar{}$	Time-averaged
${\;}_{m}$	Mixture phase
${\;}_{l}$	Liquid phase
${\;}_{v}$	Vapor phase
